# Early Social Networks Predict Survival in Wild Bottlenose Dolphins

**DOI:** 10.1371/journal.pone.0047508

**Published:** 2012-10-15

**Authors:** Margaret A. Stanton, Janet Mann

**Affiliations:** 1 Department of Biology, Georgetown University, Washington, D.C., United States of America; 2 Department of Anthropology, The George Washington University, Washington, D.C., United States of America; 3 Department of Psychology, Georgetown University, Washington, D.C., United States of America; CNRS, Université de Bourgogne, France

## Abstract

A fundamental question concerning group-living species is what factors influence the evolution of sociality. Although several studies link adult social bonds to fitness, social patterns and relationships are often formed early in life and are also likely to have fitness consequences, particularly in species with lengthy developmental periods, extensive social learning, and early social bond-formation. In a longitudinal study of bottlenose dolphins (*Tursiops* sp.), calf social network structure, specifically the metric eigenvector centrality, predicted juvenile survival in males. Additionally, male calves that died post-weaning had stronger ties to juvenile males than surviving male calves, suggesting that juvenile males impose fitness costs on their younger counterparts. Our study indicates that selection is acting on social traits early in life and highlights the need to examine the costs and benefits of social bonds during formative life history stages.

## Introduction

Although many organisms live in groups, few species have developed complex social relationships defined by features such as alliance formation, long-term individually specific relationships, and flexible group membership [1]. Socially complex species, including humans, tend to possess large, metabolically expensive brains and exhibit extended life-histories characterized by long, slow periods of growth and delayed sexual maturation [2]–[6]. If these features reflect costly correlates of complex sociality, the benefits of maintaining social bonds presumably exceed those typically associated with basic aggregation (e.g. predator protection or resource defense) [7], [8]. In recent years, research has linked alliance formation and dominance to reproductive benefits in adult males (e.g. dolphins: [9]; primates: [10], [11]) and social bonds with offspring survival and longevity in adult females (e.g. baboon: [12]–[14]; horses: [15]; rock hyraxes: [16]). Two additional studies employed quantitative genetics techniques to examine the heritability of adult social behavior, further reinforcing the connection between sociality and fitness [17], [18].

Despite the emerging empirical support for a connection between adult social bonds and fitness, the possible link between early social relationships and fitness remains unexplored. Given the flexibility and influence of early social experience [19]–[21] as well as the established relationship between ecological factors during early development and subsequent survival and reproduction [22], social conditions during early life history stages are also likely to influence fitness, particularly in species with lengthy developmental periods, extensive social learning, and early social bond-formation. Here, we employ social network analysis (SNA) to investigate the relationship between early network structure and juvenile survival in a long-lived, socially complex mammal, the bottlenose dolphin (*Tursiops* sp.).

In humans, social network features, such as the number of connections and cohesiveness of groups, are linked to lower rates of morbidity and mortality involving cardiovascular disease, cancer, and even infectious disease [23], [24]. Social relationships may encourage healthy behavior, decrease blood pressure and levels of immunosuppressive hormones, and/or serve as a stress “buffer”. These human social support studies, however, are generally interested in clinical outcomes and not survival and reproduction per se. Indeed, with the notable exception of a study predicting adolescent male social rise in a long-lived social bird (*Chiroxiphia linearis*) [25], few investigations have employed SNA to predict future fitness-related outcomes in subsequent life history stages.

Bottlenose dolphins are an excellent model for investigations into the fitness consequences of sociality since, similar to species including humans and chimpanzees, bottlenose dolphins inhabit an intrinsically complex fission-fusion social system characterized by compositionally and temporally variable groups [26]. Bottlenose dolphins also experience prolonged periods of development. In Shark Bay, Australia calves begin catching fish at 4 months of age, but are weaned at ∼3–4 years and do not reach sexual maturity until age 10–15 years [27]. Unlike primates, juvenile bottlenose dolphins are not buffered by stable kin groups between weaning and sexual maturity; therefore the post-weaning social and ecological challenges facing young bottlenose dolphins may be even greater than those facing young primates [27], [28]. Also, both male and female Shark Bay dolphins remain in their natal areas [29]; enabling both sexes to form social relationships early in life that persist into adulthood. Our previous work suggests that early social grouping patterns enable calves, particularly males, to develop social skills and bonds before the lack of social savvy might impose a fitness cost [30], [31]. Relative to females, males associate infrequently with their mothers post-weaning [29], exhibit more aggression [32], and rely on alliances for mating access [9], [33]; thus the social skills and/or bonds acquired as calves are likely to have greater fitness consequences for males than females [18].

In this study, we examine a direct fitness outcome of early social ties by testing whether any of five individual-level social network metrics (binary degree, strength, weighted betweenness, eigenvector centrality, and clustering coefficient) calculated from the networks of 67 bottlenose dolphin calves can predict juvenile survival from weaning to age 10. We did not examine social bonds during the juvenile period, only preceding it, in part because over half (57%) of the juvenile mortality occurred within a year or two after weaning resulting in an inadequate time period to reliably assess juvenile social bonds. This pattern of mortality also augments the argument that calf social patterns are critical to survival after weaning. To determine the nature of potential links between calf social bonds and future juvenile survival, we also investigated the strength of calf associations with members of each age-sex class in relation to survivorship. Given that male calves strongly associate with other male calves when separated from their mothers [31], we predicted that those male calves who survived post-weaning had stronger ties to other male calves than those who did not survive.

## Materials and Methods

### Ethics Statement

All research complies with and was approved by the Georgetown University Animal Care and Use Committee (protocol 07–041) and the West Australian Department of Environment and Conservation (license SF006897).

### Study Site and Data Collection

Data were collected as part of a long-term study of bottlenose dolphins in Shark Bay, Australia (25°47′S 113°43′E) where researchers have been investigating the life history, behavioral ecology, and genetics of the resident population since 1984 (www.monkeymiadolphins.org). The present analyses included 14,948 surveys from 1988–2010. Surveys are brief (∼5 min), opportunistic boat-based observations that represent “snapshots” of dolphin association and behavior. Group composition recorded during a survey is determined by a 10-meter chain rule in which one dolphin is considered in a group with another dolphin if they are within 10 meters of one another [34]. Individuals are identified using photographic identification based on unique dorsal fins and body markings.

### Subjects

All individuals included in this study (N_females_ = 39, N_males_ = 28) had known birth and weaning dates, were sighted on a minimum of 15 days pre-weaning (mean±sd: 67.2±53.0 total sightings; 18.36±14.90 sightings per year), and either died between weaning (∼3–4 years) and 10 years or survived past age 10. Since both sexes are philopatric [29], those individuals frequently sighted pre-weaning but not sighted for >4 years post-weaning were considered deceased. All individuals included in this study were sighted regularly by researchers and had a high probability of being sighted. Using the Cormack-Jolly-Seber model for mark-recapture data in Rcapture [35], the probability of re-sighting any individual dolphin with at least 15 sightings every four years was estimated to be (probability±sd) 0.983±0.005. Birthdates were determined by the last sighting date of the mother without a calf as well as by physical characteristics of the calf upon first sighting. Weaning dates were determined by taking a midpoint between the last date a calf was observed in infant position, from which all nursing occurs, and when association between the mother and offspring decreased to less than 50% [27].

### Social Networks

For each subject, all surveys collected during their infancy period (birthdate – weaning date) were selected, regardless of whether they included the subject, and the group composition data from this subset were used to create the full social network that that individual dolphin was part of during their infancy. Unknown or uncertain identifications were excluded (∼8% of identifications). Two dolphins were considered connected in the network if they were observed in the same group. Connections, or ties, were weighted based on a given dyad’s half weight coefficient. The half weight index is a commonly used measure of association that controls for the sighting frequency of different individuals according to the formula: X/(X+.5(Ya +Yb)+Yab) where X is the number of sampling periods (days) that A and B were observed together in the same group; Ya is the number of sampling periods A was observed without B; Yb is the number of sampling periods B was observed without A, and Yab is the number of sampling periods A and B were both observed, but in separate groups [36], [37]. In order to obtain robust measures, only individuals observed a minimum of 15 times were included in these infancy networks. Thus, each infancy network includes all members of the study population sampled at least 15 times during each subject’s infancy period, including individuals both directly and indirectly connected the subject.

### Analyses

Five social network metrics were calculated for each subject from their individual infancy network: binary degree, strength (weighted degree), weighted betweenness, eigenvector centrality, and clustering coefficient. Binary degree refers to the number of individuals directly connected to a node (number of associates); strength is the sum of the weights of all ties connected to a node; weighted betweenness quantifies how many shortest paths between two other individuals in the network pass through a node taking tie weights into account; eigenvector centrality refers to a node’s eigenvalue in the first eigenvector from a matrix of associations and is a measure of direct and indirect connectivity; clustering coefficient measures how connected a node’s neighbors are to one another and represents local network cohesiveness [37], [38]. Metrics were normalized based on the maximum value possible for a node in that network in order to compare networks of different sizes. These five metrics, as well as sex and all metric by sex interactions, served as fixed explanatory variables in a generalized linear mixed-effects model logistic regression (binomial error structure and logit link function) with individual subject, mother identity and weaning age controlled for as random variables and survival to age 10 (yes or no) as the binary response variable. Continuous explanatory variables were standardized to the same scale (standard deviation units) in order to provide a more interpretable model where one unit increase is the same for all variables, while not affecting statistical significance (Table S1). The best model was selected by Akaike information criterion (AIC) and confirmed with likelihood ratio tests. Significance of fixed explanatory variables was determined from Wald tests. Because network metrics are often correlated, multicollinearity among explanatory variables was assessed using variance inflation factors (VIFs), with VIFs greater than 10 typically considered indicative of severe collinearity [39]. VIFs in the final model ranged from 1.17–2.67.

To further investigate infancy network composition, we counted the number of each subject’s associates in each age-sex class (adult female, adult male, juvenile female, juvenile male, calf female, and calf male). Because each infancy network spans multiple years, an individual included in the network could have been observed in two age classes. In such cases, the age class with the majority of the observations was assigned to the individual. Age classes were broadly defined as: calf from 0–3.99 years, juvenile from 4–9.99 years, and adult from ≥10 years. We then summed the weights of each subject’s ties to all members of each age-sex class to get age-sex class strength. We determined whether the number of associates in each age-sex class or the strength of the relationship with each age-sex class differed between individuals who survived and those who did not using two-sample permutation tests. Separate tests were conducted for males and females. All analyses were conducted in R (version 2.13.1;R Development Core Team 2011) using the igraph package for network analysis [40], the lme4 package for GLMM logistic regression [41], and the perm package for permutation tests [42].

## Results

**Figure 1:**
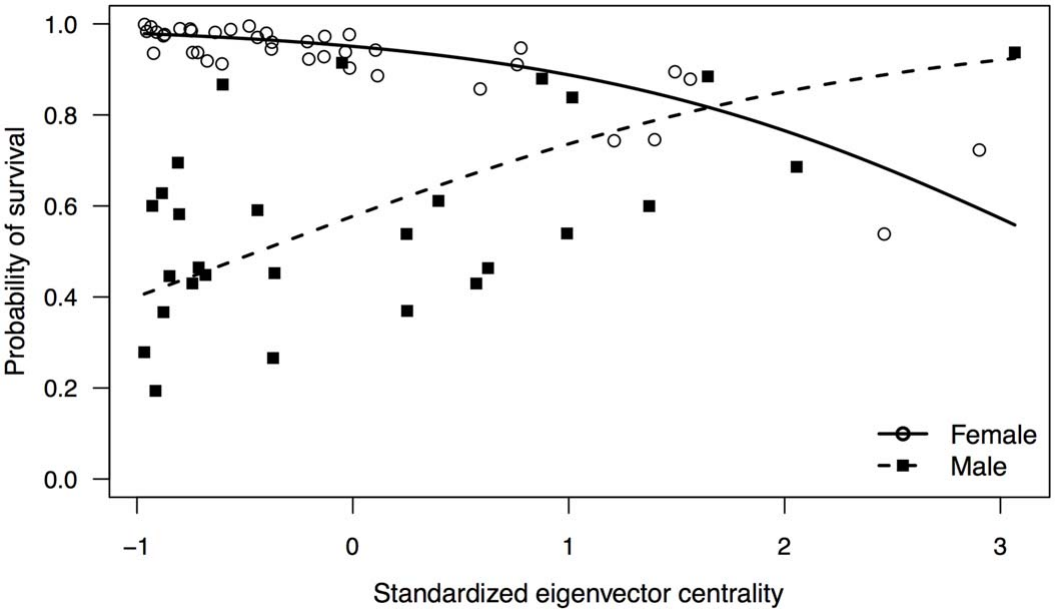
Partial effect of standardized eigenvector centrality on the probability of survival for each sex. Each point represents an individual dolphin’s (*n* = 67) probability of survival = ‘Yes’ based on their standardized eigenvector centrality as predicted by the final GLMM logistic regression model.

Males were less likely to survive from weaning to age 10 than females (Pearson’s Chi-squared test: df = 1, *χ* ^2^ = 9.6635, *P* = 0.0019). The final GLMM included the fixed effects eigenvector centrality, strength, sex, and the interaction between eigenvector centrality and sex (Table 1). The effect of eigenvector centrality on the probability of survival differed significantly between males and females as the probability of survival increased significantly with eigenvector centrality for males compared to females (Table 1; Figure 1).

**Table 1:** Parameter estimates for fixed effects in the final GLMM logistic regression model. Significance of fixed explanatory variables was determined using Wald tests.

	Estimate ± S.E.	*z*	*p*
Intercept	3.01±0.78	3.87	<0.001
Eigenvector	−0.89±0.54	−1.66	0.098
Sex	−2.65±0.87	−3.06	0.002
Strength	0.86±0.44	1.95	0.051
Eigenvector*Sex	1.60±0.74	2.17	0.030

**Figure 2:**
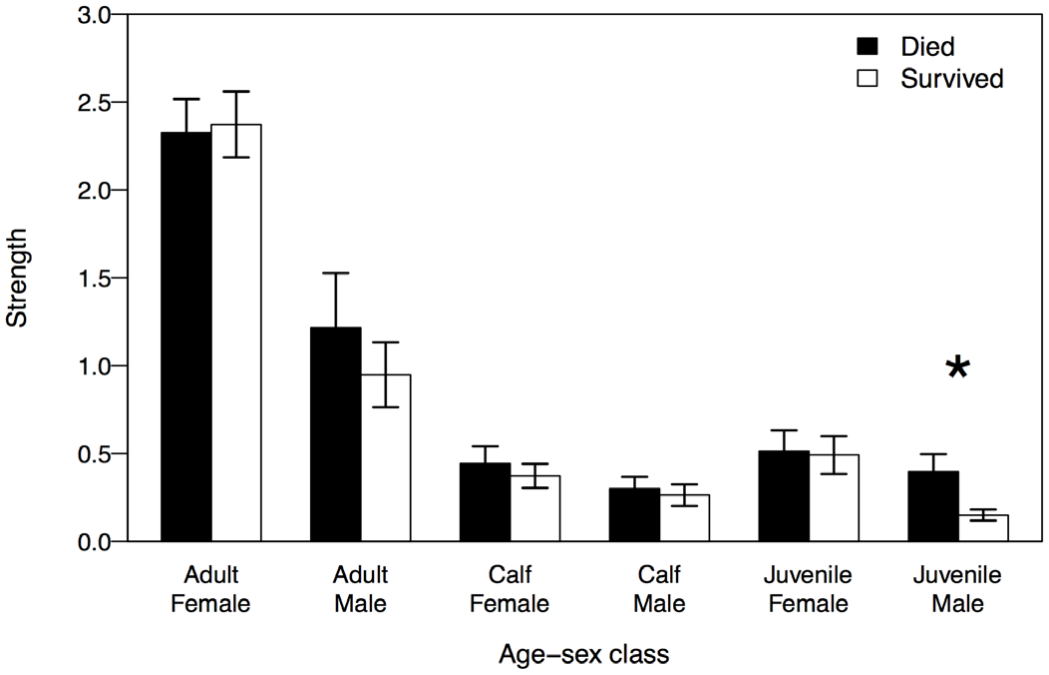
Average (±SEM) sum of associations (age-sex class strength) between male calf subjects (*n* = 28) and members of each age-sex class. Significant differences between survival outcomes are denoted by an asterisk (P<0.05).

As calves, males who died between weaning and age 10 had stronger ties to juvenile males than males who survived to age 10 (Two-sample permutation test: P = 0.016, 10,000 permutations) (Figure 2). However, calf tie strength with all other age-sex classes did not differ based on male juvenile survival status. Age-sex class strength also did not differ significantly between female calves based on survival status and the number of associates belonging to each age-sex class did not significantly differ among either sex calves based on survival status.

## Discussion

Differential survival between males and females is common across mammals [43], and our results are consistent with the observation that males are typically less likely to survive the juvenile period. However, this study identifies a unique social component to the likelihood of survival. A growing body of evidence indicates that social bonds and social capital are beneficial for social mammals; particularly regarding lower levels of stress hormones, which may in turn influence immune response [44]–[46]. The interaction with eigenvector centrality is particularly notable because this metric accounts for both direct and indirect ties and is therefore not detectable using non-network techniques. That binary degree, or number of associates, was not a significant predictor of survival, suggests that when considering social partners, quality is more important than quantity, and argues against the practice of using group size as a sole indicator of social complexity. Individuals with high eigenvector centrality are either themselves central to the network, or are connected to central individuals [47]. In this case, calves’ strong connections to their mothers are likely driving eigenvector centrality since mothers were present in the majority of the surveys and mothers had social network metric values similar to those of their offspring, thus we cannot determine the degree to which the mother or the calf is responsible for the their local network structure. Eigenvector centrality values calculated excluding maternal association did not significantly differ from those including maternal association (Two-sample permutation test: P = 0.274, 10,000 permutations). Maternal social choices clearly influence the social environment experienced by calves, however, in this analysis we controlled for maternal identity and still found that infancy networks were predictive of survival during the juvenile period, when offspring have markedly decreased maternal association [29].

Evidently, the nature and quality of social bonds is more important than the number of associates. While our earlier results show that early social ties have benefits, juvenile males are likely to be a source of social stress for male calves and detrimental to male calf fitness. The harmful effects of chronic social stress are well documented, particularly in rodents and non-human primates where social stress affects abnormal aggressive behavior, organ weight, plasma glucocorticoid levels, body weight, fat distribution, insulin production, testosterone levels, the dopamine and serotonin systems, and even hippocampal neuronal morphology [48]. We suggest that juvenile males directly harass their younger counterparts. In a previous investigation of socio-sexual interactions among Shark Bay bottlenose dolphins, male calves were the recipients of 49.4% of all (primarily mounting) events with juvenile male actors. Female calves received just 23.6%, less than half as many events as their male counterparts. Conversely, juvenile males were recipients of only 4.8% of socio-sexual events with male calf actors, while other male calves received 42.3% of these events [49]. Larger juveniles do not appear to be playfully self-handicapping in these interactions by allowing the smaller calves to mount them, suggesting that juvenile male mounting of male calves is about dominance and intimidation rather than affiliation. Juvenile males might be decreasing the fitness of their future competitors, and male calves could be easy targets, particularly those with mothers less central in the overall social network. Precisely how maternal and calf associates might protect male calves from juvenile harassment is not yet known. Finer scale investigations are needed to tease out the significance of these relationships as well as the mother’s role.

Recent evidence suggests three levels of alliances in adult male bottlenose dolphins, a level of complexity yet to be demonstrated in any species besides humans [50]. Given the intense competitive nature of male relationships in this context, it follows that establishing bonds or at least some form of social competency prior to reaching adulthood would benefit males in negotiating this challenging social landscape. Our results suggest that early social bonds or skills established as calves provide males with either a competitive advantage or a social buffer post-weaning when maternal protection is no longer available. Since adult female bottlenose dolphins do not form alliances, the consequences of early sociality appear to differ from those of their male counterparts. As in baboons and horses, female social bonds in bottlenose dolphins likely impact reproductive success rather than survival per se [18].

Overall, our results demonstrate the significance of early social bonds in a socially complex mammal. Previous investigations demonstrated the adaptive value of adult social relationships; however many socially complex species have delayed maturation, spending a relatively large portion of their life span as dependent offspring and/or juveniles. Prior to reproduction, bottlenose dolphins spend approximately a decade navigating a dynamic, multi-level social environment. The ability to predict juvenile survival from calf social network metrics suggests that selection is acting on social traits at these early life-stages. The potential fitness consequences of social traits at all life stages must be investigated and accounted for in order to fully understand the evolution of sociality and the causes and consequences of social complexity.

## Supporting Information

Table S1 Standardized network metrics for each calf (*n* = 67).
(DOCX)
