# Supplementary material for: Early Social Networks Predict Survival in Wild Bottlenose Dolphins
Source: PLoS One. 2012 Oct 15;7(10):e47508. doi: 10.1371/journal.pone.0047508 (PMC3471847; doi:10.1371/journal.pone.0047508)
Supplement: Table S1 — Standardized network metrics for each calf (n = 67). (DOCX) [file pone.0047508.s001.docx]

**Table S1**

| **Calf ID #** | **Mother ID #** | **Survived to 10** | **Eigenvector** | **Binary degree** | **Strength** | **Betweenness** | **Clustering coefficient** |
| --- | --- | --- | --- | --- | --- | --- | --- |
| 1 | 1 | Y | -0.442 | -0.190 | 0.369 | -0.864 | -0.333 |
| 2 | 2 | N | 1.644 | -0.712 | 0.577 | -0.584 | -0.091 |
| 3 | 30 | Y | -0.743 | 0.668 | -0.134 | 1.583 | 1.769 |
| 4 | 6 | Y | -0.966 | -0.040 | -0.722 | -0.399 | -0.379 |
| 5 | 4 | Y | 0.876 | -0.731 | 1.156 | -0.544 | -0.888 |
| 6 | 17 | Y | -0.363 | -1.345 | -0.342 | -0.425 | -0.913 |
| 7 | 20 | Y | 0.779 | -0.931 | 0.652 | -0.250 | 0.030 |
| 8 | 23 | Y | -0.714 | 1.293 | 0.006 | -0.869 | -1.800 |
| 9 | 41 | N | -0.683 | -1.658 | -0.096 | -0.619 | -0.372 |
| 10 | 18 | N | -0.804 | 0.893 | 0.626 | 0.960 | -0.012 |
| 11 | 12 | Y | 2.056 | 0.046 | -1.216 | -0.773 | -0.483 |
| 12 | 3 | Y | -0.871 | -0.079 | -0.059 | -0.733 | 0.631 |
| 13 | 5 | N | -0.914 | -0.829 | -1.314 | -0.447 | -0.123 |
| 14 | 8 | Y | 0.992 | -1.051 | -1.057 | 2.125 | 0.267 |
| 15 | 25 | Y | -0.204 | -0.798 | -0.824 | -0.614 | 1.635 |
| 16 | 15 | Y | -0.606 | 0.984 | -1.401 | -0.359 | -0.985 |
| 17 | 10 | N | 0.249 | 1.263 | -0.448 | -0.658 | 0.878 |
| 18 | 9 | Y | -0.674 | 0.295 | -1.370 | 0.337 | 1.551 |
| 19 | 10 | Y | -0.133 | -1.086 | -0.663 | 0.135 | -0.933 |
| 20 | 11 | Y | -0.752 | 0.883 | 0.924 | 0.098 | 0.078 |
| 21 | 12 | N | 0.590 | 0.786 | -0.803 | -0.258 | 0.464 |
| 22 | 43 | Y | -0.377 | -1.055 | -0.590 | 0.556 | -0.168 |
| 23 | 14 | Y | -0.441 | -1.472 | 0.093 | 0.319 | -0.302 |
| 24 | 33 | Y | -0.935 | 1.690 | 1.323 | -0.832 | -1.782 |
| 25 | 18 | N | -0.369 | 0.250 | -1.291 | -0.591 | -0.761 |
| 26 | 16 | Y | -0.014 | -0.422 | -0.914 | -0.844 | -1.445 |
| 27 | 17 | N | -0.877 | -0.768 | -0.328 | -0.769 | -1.447 |
| 28 | 24 | N | -0.849 | -0.583 | 0.030 | 2.030 | 0.408 |
| 29 | 7 | Y | -0.800 | 1.602 | 0.965 | 0.066 | 0.373 |
| 30 | 27 | Y | -0.955 | 1.918 | 0.266 | -0.882 | 1.018 |
| 31 | 18 | N | 0.572 | 1.074 | -1.221 | -0.093 | -1.213 |
| 32 | 18 | Y | 1.373 | 0.039 | -1.088 | 2.000 | 0.425 |
| 33 | 19 | Y | -0.929 | 0.623 | 0.817 | -0.817 | 1.896 |
| 34 | 29 | Y | 1.398 | 0.029 | -0.801 | 1.208 | -1.711 |
| 35 | 20 | Y | -0.719 | -1.179 | -1.100 | -0.773 | 0.400 |
| 36 | 36 | Y | 1.563 | -0.432 | 0.416 | 0.088 | -1.164 |
| 37 | 38 | Y | -0.034 | 0.126 | -0.373 | 1.767 | -0.875 |
| 38 | 26 | Y | -0.603 | -0.687 | 2.244 | 0.458 | 0.509 |
| 39 | 22 | Y | -0.129 | -0.897 | 0.503 | -0.165 | -1.531 |
| 40 | 21 | Y | -0.810 | 1.425 | 1.202 | 0.566 | -1.369 |
| 41 | 24 | N | 0.252 | 0.082 | -1.247 | -0.528 | 0.025 |
| 42 | 34 | Y | -0.748 | -0.194 | 0.592 | -0.706 | -0.791 |
| 43 | 12 | Y | -0.480 | 1.176 | 2.282 | -0.367 | 0.421 |
| 44 | 29 | Y | -0.909 | 1.212 | 0.206 | -0.540 | 0.410 |
| 45 | 2 | Y | 0.105 | -0.446 | -0.139 | -0.223 | 2.675 |
| 46 | 28 | Y | 1.017 | -0.033 | 0.644 | -0.884 | 0.934 |
| 47 | 24 | N | 0.396 | 0.356 | -0.225 | -0.059 | 0.886 |
| 48 | 34 | Y | 1.492 | 1.459 | 0.527 | -0.824 | -0.650 |
| 49 | 4 | Y | -0.637 | -0.696 | 0.438 | -0.244 | -0.481 |
| 50 | 37 | N | -0.884 | -1.573 | 0.916 | -0.545 | 0.135 |
| 51 | 31 | N | 2.461 | -0.920 | -0.776 | 1.490 | -0.546 |
| 52 | 13 | Y | -0.923 | 1.707 | -1.333 | -0.776 | 1.267 |
| 53 | 35 | Y | -0.877 | -1.040 | -0.192 | -0.592 | 0.770 |
| 54 | 40 | Y | -0.566 | -0.404 | 1.012 | 1.157 | 0.066 |
| 55 | 32 | Y | -0.401 | 0.074 | 0.591 | -0.072 | -0.553 |
| 56 | 44 | Y | -0.050 | 0.705 | 2.363 | 0.743 | -1.205 |
| 57 | 35 | Y | 0.626 | 0.179 | -1.108 | -0.001 | 1.746 |
| 58 | 27 | Y | -0.964 | 1.816 | 3.142 | -0.754 | 0.809 |
| 59 | 28 | N | -0.375 | 1.951 | -0.189 | -0.593 | -0.011 |
| 60 | 39 | Y | 0.114 | 0.006 | -0.991 | -0.205 | 0.472 |
| 61 | 40 | Y | 3.067 | -1.073 | 0.173 | -0.374 | 0.748 |
| 62 | 12 | Y | -0.211 | -0.431 | 0.016 | -0.410 | -0.159 |
| 63 | 43 | Y | 0.762 | -0.899 | -0.017 | 2.498 | -1.122 |
| 64 | 42 | Y | -0.742 | -1.832 | -1.116 | 0.137 | 0.643 |
| 65 | 40 | Y | 1.210 | 0.132 | -1.009 | 4.188 | 0.252 |
| 66 | 46 | Y | -0.016 | 0.972 | 0.812 | -0.829 | 0.093 |
| 67 | 45 | Y | 2.902 | -1.229 | 0.611 | -0.824 | 1.918 |
